# Re-entry Adjustment and Job Embeddedness: The Mediating Role of Professional Identity in Indonesian Returnees

**DOI:** 10.3389/fpsyg.2018.00792

**Published:** 2018-05-28

**Authors:** Sonny Andrianto, Ma Jianhong, Confidence Hommey, Devi Damayanti, Honey Wahyuni

**Affiliations:** ^1^Department of Psychology and Behavioral Sciences, Zhejiang University, Hangzhou, China; ^2^Department of Psychology, Islamic University of Indonesia, Yogyakarta, Indonesia; ^3^Department of Psychology, Ahmad Dahlan University, Yogyakarta, Indonesia

**Keywords:** Indonesian returnees, re-entry adjustment, professional identity, job embeddedness, demographic characteristics

## Abstract

The present study examined the relationship between difficulty in re-entry adjustment and job embeddedness, considering the mediating role of sense of professional identity. The online data on demographic characteristics, difficulty on re-entry adjustment, sense of professional identity, and job embeddedness were collected from 178 Indonesian returnees from multiple organizations. The results showed that difficulty in re-entry adjustment was a significant predictor of a sense of professional identity; a sense of professional identity was a significant predictor of job embeddedness. Furthermore, sense of professional identity is an effective mediating variable, bridging the relationship between post-return conditions to the home country and work atmosphere. Finally, the key finding of this study was that sense of professional identity mediated the effect of difficulty in re-entry adjustment on job embeddedness. The theoretical and practical implications, study limitations, and future research needs of our findings are noted.

## Introduction

Becoming an international student is the dream of many young adults in many countries. The United Nations Educational, Scientific, and Cultural Organization (UNESCO) defines international students or internationally mobile students as individuals who have crossed a national border for the purpose of education and are now enrolled outside their home country (UNESCO, 2012). In line with this, the Organization for Economic Co-operation and Development (OECD) reported that in 2011, nearly 4.3 million students were enrolled in university-level education outside their home country, and 53% of them were Asian students; China, India, and Korea having the highest numbers (OECD, 2013).

Most countries around the world undertake efforts to support students and citizens in general to study in specific fields that are growing rapidly in their home country, as well as to gain some amount of international experience, placing them a little above the bar compared to those who do not have such an opportunity. Studying abroad mainly is an opportunity to access better quality education, experience in academic atmosphere, and acquire knowledge and skills that may not be obtained in the home country (Mayrhofer et al., 2004; Arnaez et al., 2014; Shin et al., 2014; Kent-Wilkinson et al., 2015; Cuellar, 2016; Forbush and Foucault-Welles, 2016; Petersdotter et al., 2017).

The advantages of studying abroad also come in the form of development of cultural sensitivity, personal challenge, personal and professional development, and other personal and mental improvement to compete on a global level (Salisbury et al., 2009; Eder et al., 2010; Goel et al., 2010; Presley et al., 2010; Molony et al., 2011; Salisbury, 2011; Ruble and Zhang, 2013; Zhuang et al., 2015; Kelleher et al., 2016). On the other hand, upon returning to the home country, returnees often feel that the cultural values, symbols, behaviors, and rules that they assimilated while in the foreign country do not fit or sometimes conflict with what is accepted in their own home country (Gama and Pedersen, 1977; Martin, 1984; Uehara, 1986; Moore et al., 1987; Seiter and Waddell, 1989). Irrespective of this, Indonesia must avail itself of the prevailing knowledge, skills, and experiences propelling development in other countries.

Indonesia is the most populous country in the world and according to the 2010 national census, the population of Indonesia is 237.6 million (Statistics Indonesia [BPS], 2017). With a population growth at 1.9% and according to the projection of the United Nations by looking at the absolute population of Indonesia in the future, it will have a population of over 270 million in 2025, over 285 million in 2035, and 290 million in the year 2045 (United Nations, Department of Economic and Social Affairs, Population Division, 2015). The Indonesian government is very much concerned about education as the constitution stipulates that 20 percent of the national budget is to be prioritized for education (Ministry of Finance of Republic of Indonesia, 2016). In 2015, there were approximately 4.3 million Indonesian citizens living abroad, most of them overseas workers (60%), followed by international students (20%), and the others are ship crew, housewives, diaspora, etc. (Statistics Indonesia [BPS], 2017).

There is limited information and empirical research available on Indonesian citizens who returned to the country after completing their study or assignment overseas. Further, information and empirical research available on the impact of the difficulty of re-entry adjustment on professional identity and the organizational field in general are limited. The arrival of organizational members after completing studies abroad, in addition to raising new expectations, is also followed by worries. The worries and criticism voiced by organizational members and administrators who have respect for their organization sounds alarming with regards to organizations losing their potential members.

Consequently, the following questions were addressed: (1) Is re-entry an easy transition? (2) Do returnees’ characteristics and time dimensions affect the re-entry adjustment process? (3) Does the knowledge, expertise, and skills that were gained overseas make one feel more professional on the job? (4) Does the experience gained overseas have an effect on the home country organization when they return to their home country? As such, it is important to examine simultaneously how re-entry adjustment and perceived professional identity are related to each other, and how both are related to job embeddedness. Thus, the present study aims at filling the above gaps in the empirical study by addressing how difficulty in re-entry adjustment, along with professional identity, affects job embeddedness.

Gaw (2000) and Maybarduk (2008) reported that the first publication about re-entry phenomena was published by Alfred Scheutz in 1945. He observed the veteran of World War II then used the term *homecomer*, as someone who had difficulty when they returned home, especially in social relationships with family and friends and cultural adaptation with the society they had long left behind (Maybarduk, 2008). However, re-entry experiences can also present “sweet” sides: when returnees re-encounter the people and the cultural habit of their home country, as well as when they have an exchange of thoughts and feelings with those who also share the experience of intercultural interaction (Kartoshkina, 2015). In this study, we focused on the “bitter” side of re-entry adjustment experiences.

The phenomenon of the “bitter” side in a number of previous researchers has been seen in the light of re-entry culture shock, reverse culture shock, or re-entry shock, in which all three concepts share almost identical meaning, and that is how the difficulty of re-entry adjustment experienced by the returnee is described after staying overseas for a certain period of time. Those can be described as a period when the sojourner might be facing psychosocial difficulties and/or physical problems when they return to their home country after living abroad for a while (Uehara, 1986). Furthermore, Gaw (2000) argued that reverse culture shock can be viewed as having the same definition as culture shock, but reverse culture shock focuses more on difficulties in re-adjusting, re-acculturating, and re-assimilating for returnees in their own home culture after living in a different culture for a period of time.

Seiter and Waddell (1989) stated that re-entry shock is seen in the difficulty in readjusting to the returnees’ own culture and occur when returnees are back to their home country and discover or feel that their friends, family, and colleagues have changed and no longer match with their current mental image. The implication being that besides cultural conflict between host culture and home culture, pressure and high expectation from home culture, i.e., work environment, family, and colleagues, are also causes of re-entry shock. Freedman (1986) explains that to feel comfortable, a person must be able to predict the behavior of others. After returning from abroad, returnees face pressure and more expectation from home culture, which was previously unpredictable. This pressure, person–organization expectation gap, and person–community expectation gap can result in increased stress and uncomfortable feeling which is a part of re-entry shock (Seiter and Waddell, 1989; Shen and Hall, 2009).

Through the demographic characteristics of the returnees, we can learn a lot about the differences in demographics that affect their re-entry adjustment (Szkudlarek, 2010). Gender, age, marital status, and educational level are some of the characteristics returnees that influence the process of repatriation. Studies conducted by Rohrlich and Martin (1991) and Francees (2012) showed that females easily readjust and feel more comfortable when they return to the home country than the males. Age difference has an interconnection with re-entry shock, where the readjustment for younger returnees will be harder when they return to their home country (Martin, 1984; Black and Gregersen, 1991; Hammer et al., 1998; Cox, 2004; Francees, 2012; Soon, 2012; Ha and Jang, 2015; Kartoshkina, 2015). Some studies have found that marital status of returnees also reflect on re-entry shock, such that returnees who are single reported to have more problems and distress in re-entry adjustment compared to married returnees (Cox, 2004; Francees, 2012; Soon, 2012).

Moreover, some previous studies also examined the dimensions of time as a factor affecting a successful re-entry adjustment process. The length of time living overseas influences the adaptation process when one returns to the home country; the longer the period of life overseas, the more difficult re-entry adjustment feels (Cox, 2004; Vidal et al., 2007; Francees, 2012; Soon, 2012; Kartoshkina, 2015), especially in the context of work (Black and Gregersen, 1991). Furthermore, the length of time one stays in the home country after re-entry can also be used as a predictor in explaining the process of re-entry adjustment (Szkudlarek, 2010). Re-entry shock is often felt in the early stages of returnees’ re-entry adjustment process to the home country (Black and Gregersen, 1991; Gregersen and Stroh, 1997; Francees, 2012; Soon, 2012).

Professional identity is an essential construct in understanding persons’ professional lives and their future career. Understanding of professional identity is linked to professional learning, personality traits, and professionalism (Hoyle and John, 1995). Professional learning gained from the process of learning and experience gained during their practice of the profession aids in understanding professional identity. Professionalism can be realized through the transformation of self-concept into creativity, evident in professional work and activity (Hoyle, 1981; Davies, 2013).

Avraham (as cited in Fisherman, 2015) argued that the concept of professional identity include the perception of persons’ qualities, talents, feelings, professional values, and interactions with other people in their profession. In a simpler way, Paterson et al. (2002) define professional identity as the sense of being a professional. Furthermore, assumptions regarding professional identity as raised by Rodgers and Scott (2008), are built and depend on a range of contexts, established via relations with others, and strive to be coherent. In this vein, we espouse a view that professional identity is a subjective personal identification of the capacity and capability of people in the profession they are engaged with.

Professional identity continues to evolve involving professional practice, talent development, and the values of the profession (Adams et al., 2006). It should be noted that there is a difference between the idealized version of the profession and the real work practiced by the existing members of the profession (Cohen, 1981; Melia, 1987). For the novice professional, it is necessary to establish an identity as a professional through thinking, experience, and critical understanding of the current profession, as well as the existence of role models (Adams et al., 2006). Thus, understanding professional identity is important for gaining insight into aspects of professional lives such as personal lives, emotion, future career, and commitment to the job or profession.

Gu and Schweisfurth (2015) state that the experiences of returnees while studying abroad and returning home make an actual impact on their professional identity and personal lives. Someone staying and participating in activities when overseas connotes a contribution to the development of professional expertise and a better understanding of the global organization (Hocking et al., 2007). The final phase of professional development is the outcome: professional identity is the primary product in addition to specific technical competencies (Watts, 1987). Unfortunately, there has been very limited empirical research on investigating the impact of re-entry adjustment to professional identity. Thus, it is important to address in this study the Indonesian returnees’ perceived professional identity change resulting from their international educational experiences. Thus, we predict:

*Hypothesis 1*: Success in re-entry adjustment process will be positively associated with increase in returnees’ sense of professional identity.

The construction of job embeddedness was first raised by Mitchell et al. (2001) as a critical factor in understanding why people stay on their jobs. Until 2012, job embeddedness theory was still seen as a relatively new perspective in research related to the world of work, more specifically on turnover (Zhang et al., 2012). Job embeddedness is one of the specific elements that bind individuals in the organization. Job embeddedness can be analogized as a net where one can be trapped and is one of the key factors in understanding why individuals decide to stay or quit their jobs (Mitchell et al., 2001; Shen and Hall, 2009).

Mitchell et al. (2001) reveal three dimensions that reflect important aspects of job embeddedness: Link, Fit, and Sacrifices. Furthermore, these three dimensions are categorized into two sub-dimensions, the organization and their communities, making the dimensions of job embeddedness six in total (Mitchell et al., 2001; Crossley et al., 2007; Zhang et al., 2012).

Links can be defined as formal or informal connections between persons, institutions, or other people in their social community (Mitchell et al., 2001; Lee et al., 2004; Zhang et al., 2012). This understanding indicates that link does not only refer to the quantity of social bonding that exists but also the quality of the psychological relationships that are intertwined with the people at the workplace and outside the workplace. The fit is interpreted as the compatibility and comfortable feeling of the employees to the conditions of the organization where they work, and with the community outside of their workplace (Mitchell et al., 2001; Zhang et al., 2012). A person who has a fit feature is a person who has a match between his personal value and the organizational culture and can adapt to his social community, while sacrifice is a consequence of material, psychological, and social cost that has the potential to be paid or lost when someone leaves the organization or community (Mitchell et al., 2001; Zhang et al., 2012).

Little research was available examining the relationship between job embeddedness and demographic characteristics. The results of those studies showed that female employees (Curtin and Wilkes, 2007; Peltokorpi, 2013) and younger employees (Curtin and Wilkes, 2007) are less embedded in the organization for various reasons. Other studies place demographic characteristic only as a descriptive exposure of the distribution of research subjects.

Professional identity, as a series of studies on social identity notes, can be interpreted as the extent to which individuals perceive their professional role as essential, attractive, and aligned with other roles (Hofman, 1977, 1985; Moore and Hofman, 1988). Social identity itself can be interpreted as a person’s sense of whom they are based on their group membership and sense of belonging to their group (Tajfel and Turner, 1986). In the perspective of social identity theory, professional identity focuses on group interaction in the work environment and also relates to how individuals compare and distinguish themselves with other professional groups (Adams et al., 2006).

Professional enhancement and personal experience often bring about external job opportunities (Cheng, 2014). Undeniably, individuals will move from one organization to another to pursue the best opportunities for their professional development (Lazarova and Caligiuri, 2001). On the other hand, the job embeddedness theory states that if an employee feels compatible or comfortable with their organization environment, they will feel professionally and personally tied to the organization (Mitchell et al., 2001). Based on some of these opinions, we assume that professional identity can be a predictor of a person’s decision to embedded in their organizations. From a role identity perspective, it may be that positive sense of professional identity being individuals to embedded in their current profession’s organization, and therefore, the study proposes to test the following hypothesis:

*Hypothesis 2*: Increase in returnees’ sense of professional identity will be positively associated with an increase in job embeddedness.

Re-entry adjustment represents the psychological process experienced by returnees when returning to the home country (Seiter and Waddell, 1989). Returnees’ challenges associated with repatriation issues and re-entry process reflects a lack of commitment to stay in their home-country organization (Szkudlarek, 2010). On the other hand, job embeddedness represents the conceptual and perceptual forces that keeps a person on his or her current job (Crossley et al., 2007). Job embeddedness is one of the crucial indicators of organizational success during the international assignment, that is, the specific elements that bind individuals to the organization and success indicator of the international assignment process (Shen and Hall, 2009).

Repatriation is a censorious phase in returnees’ careers; when they re-establish their professional identity within the home country organization (Lazarova and Tarique, 2005). We believe that a sense of professional identity may play a mediating role in the effect of reentry adjustment process on job embeddedness. Furthermore, we posit that the more successful re-entry adjustment is, the higher the likelihood that an employee will feel professionally and personally tied to the home country organization.

In an attempt to investigate the proposition made, we posit in the conceptual model that the effect of having it difficult or easy in re-the entry adjustment process may also be indirectly related to job embeddedness through the mediating effects of sense of professional identity. Considering arguments raised in hypotheses one and two, we propose a formal relationship in hypothesis 3:

*Hypothesis 3*: The relationship between job embeddedness and difficulty in re-entry adjustment will be mediated by perceived professional identity.

A number of studies place the time dimension as a predictor of success or failure of the re-entry adjustment process. Repatriates who spend much of their time abroad tend to be have more difficulty in the process of readjustment upon returning home (Black and Gregersen, 1991; Black, 1992; Forster, 1994; Gregersen and Stroh, 1997; Suutari and Välimaa, 2002). Identical to the length of time living abroad, the time elapsed since the arrival back home is also a predictor of the re-entry adjustment process (Gregersen and Stroh, 1997; Vidal et al., 2007). To control any possible effects of the length of stay abroad and length of stay back in the home country on Hypothesis 3, they were both selected as theoretical covariates.

## Materials and Methods

### Participants

All participants in this study were Indonesian returnees who have a job and just completed their study or job assignment overseas and have returned to Indonesia. As much as 244 online questionnaires were distributed through email to all potential participants. A totla of 76 potential participants were eliminated among the sample due to incomplete responses and not having a job yet, and as such 178 responses were used for further analysis.

### Procedures

Data were collected through an online survey. Based on a list of Indonesian returnees we already have, we sent an e-mail to the potential participants inviting them to participate in this study. After that, we only considered participants who currently have jobs.

The online survey contained two main parts: an introduction and a number of research scales. In the introductory section, we described the identity of the researchers, research objectives, research procedures, and confidentiality undertakings of the participants. Informed consent statements were explained into separate screen appear before the participants can access the survey by click on an “I agree” button, as recommended by Schmidt (1997). The section containing the research scale consisted of demographic profiles and four questionnaires to uncover re-entry adjustment, professional identity, and job embeddedness. All aspects and items in the online survey were written in the Indonesian language.

For all questionnaires, back translation methodologies were used (Brislin, 1970). First, an Indonesian research assistant translated all questionnaires from English into Bahasa (Indonesia’s national language). Second, another Indonesian research assistant, who is an expert in the English language, translated the Bahasa version back to English. Finally, both versions were compared and corrected until the second English version matched very closely with the original English version. The study was approved by the Medical and Health Research Ethical Committee of the Faculty of Medicine, Islamic University of Indonesia, and was carried out in accordance with the Declaration of Helsinki.

The coefficient of Cronbach’s alpha was used to assess the reliability of the measures of all scales in this study. Following Kline (as cited in Neto et al., 2016), the reliability coefficient of the measures ranged between 0.70 and 0.90, which reflects a good category, and 0.91 and 0.99, which reflects a perfect category.

### Instruments

*Re-entry adjustment*. Re-entry adjustment after returning to the home country was measured using a Re-entry Shock Scale by Seiter and Waddell (1989), which had a coefficient of internal consistency in terms of Cronbach’s alpha score of 0.830. The original form of RSS was compiled based on previous research (e.g., Church, 1982; Martin, 1984; Sussman, 1986; Uehara, 1986). This scale consists of 16 items and measured using a 7-point Likert scale ranging from 1 (Strongly disagree) to 7 (Strongly agree). Sampled items for Re-entry Shock Scale are: *Life was more exciting in the host culture*, and *When I returned home I felt really depressed*. The higher the score obtained, the more difficult the participants interpreted the process of adaptation to be when returning to their home country, that is, the greater the sense of reverse culture shock. For this study, the overall reliability, Cronbach’s alpha score for the scale was 0.802 with the corrected item-total correlation between 0.271 and 0.570.

*Professional identity*. We adapted the Professional Identity Scale developed by Adams et al., (2006) to measure social identity; it reflects group interactions in the workplace and relates to how people compare and differentiate themselves from other professional groups. Professional Identity Scale consists of 9 items in one-dimension solution and responses were obtained on a 5-point Likert scale, ranging from 1 (strongly agree) to 5 (strongly disagree), where higher scores indicate a stronger professional identity and lower scores a weaker professional identity. Sampled items for Professional Identity Scale are: *I find myself making excuses for belonging to this profession*, and *I can identify positively with members of this profession*. In this study, the overall reliability, Cronbach’s alpha score for the scale was 0.769 with the corrected item-total correlation between 0.329 and 0.745.

*Job embeddedness*. Measuring job embeddedness, we adopted the Global Job Embeddedness Scale developed by Crossley et al. (2007). Global Job Embeddedness Scale is constructed to reveal work and non-work factors together to explain the individual’s reasons to embed in their current jobs or organizations. The Global Job Embeddedness Scale consisted of 7 items with a single-dimension solution and rated on a 5-point agreement scale from 1 (strongly disagree) to 5 (strongly agree). Sampled items for Global Job Embeddedness Scale are: *I feel attached to this organization*, and *I feel tied to this organization*. The reliability, Cronbach’s alpha score of the scales in this study was 0.853 with the corrected item-total correlation between 0.372 and 0.733.

*Demographic variables*. We examined the unique effects of returnees’ demographic variables such as gender (male = 1, female = 2), age (30 years or below = 1, between 31 and 40 years = 2, above 40 years = 3), education level (bachelor’s degree = 1, master’s degree = 2, doctor’s degree = 3), and marital status (single = 1, married = 2). We also controlled the length of stay abroad (12 months or less = 1, between 13 and 24 months = 2, more than 24 months = 3) and the length of stay back in the home country (12 month or less = 1, between 13 and 24 months = 2, more than 24 months = 3) as factors affecting re-entry adjustment to job embeddedness via professional identity.

## Results

### Demographic Profile and Correlations

**Table 1** shows demographic information on gender, age, educational level, marital status, the length of returnees’ stay overseas, and length of time they’ve stayed back in the home country. Among the sampled participants, 70 (39.3%) were male and 108 (60.7%) were female. Majority of the participants were within the age of 21–30 years (67.5%), and the rest were younger than 21 years (1.1%) and older than 30 years (31.4%). Most participants reported being married (60.7%) and 82% of the participants had attained a master degree.

**Table 1:** Demographic table of participants.

Profile	Groups	Frequency	Percentages
Gender	Male	70	39.3
	Female	108	60.7
Age	≤30	122	68.5
	31–40	42	23.7
	>40	14	7.8
Educational level	Bachelor’s degree	2	1.1
	Master’s degree	146	82
	Doctorate degree	30	16.9
Marital status	Single	108	60.7
	Married	70	39.3
Length of stay abroad	≤12 months	72	40.4
	13–24 months	68	38.2
	>24 months	38	21.4
Length of stay back	<12 months	116	65.2
home country	13–24 months	40	22.5
	>24 months	32	12.3

All of the participants at the time of data collection were living in Indonesia and those who had the experience of living overseas for less than one year were of majority (40.4%), 1–2 years (38.2%), and 21.4% have lived in overseas for more than 2 years. The demographic distributions relating to the continent participants lived while overseas were Europe (75.3%), Australia (13.5%), and Asia (11.2%). When this study was conducted, those who had settled back in Indonesia for less than one year formed the majority (65.2%) and the others (34.8%) are more than 1 year.

**Table 2** shows the means, standard deviations, and inter-correlations among the three primary variables and demographic characteristic of returnees. With regards to returnees’ demographic variables, re-entry shock correlated with gender (*r* = 0.345; *p* < .01), age (*r* = -0.313; *p* < 0.01), educational level (*r* = -0.256; *p* < 0.01), marital status (*r* = -0.366; *p* < 0.01), and length of stay back in home country (*r* = -0.450; *p* < 0.01). Professional identity correlated with age (*r* = 0.224; *p* < 0.01), educational level (*r* = 0.317; *p* < 0.01), marital status (*r* = 0.415; *p* < 0.01), and length of stay back in home country (*r* = 0.159; *p* < 0.05). Job embeddedness correlated with age (*r* = 0.380; *p* < 0.01), educational level (*r* = 0.353; *p* < 0.01), marital status (*r* = 0.243; *p* < 0.01), length of stay overseas (*r* = 0.288; *p* < 0.01), and length of stay back home (*r* = 0.269; *p* < 0.01).

**Table 2:** Descriptive statistics and inter-correlations among variables (*N* = 178).

Variables	1	2	3	4	5	6	7	8	9
1. Gender	1								
2. Age	-0.170*	1							
3. Educational level	-0.087	0.748**	1						
4. Marital status	-0.247**	0.564**	0.379**	1					
5. Length stay overseas	-0.051	0.564**	0.484**	0.182*	1				
6. Length stay back home	-0.072	0.455**	0.269**	0.335**	0.158*	1			
7. Re-entry shock	0.345**	-0.313**	-0.256**	-0.366**	-0.093	-0.450**	1		
8. Professional identity	0.068	0.224**	0.317**	0.415**	0.058	0.159*	-0.287**	1	
9. Job embeddedness	-0.067	0.380**	0.353**	0.243**	0.288**	0.269**	-0.133	0.194**	1
Mean value	1.61	3.20	2.16	1.39	2.93	2.03	51.506	38.234	24.528
SD	0.490	1.346	0.395	0.490	1.061	1.225	11.197	4.490	6.041

*^∗^Correlation is significant at 0.05. ^∗∗^Correlation is significant at 0.01.*

Meanwhile, between the three main variables, we realized that re-entry shock correlated significantly and negatively with professional identity (*r* = -0.287; *p* < 0.01); that means the result supported Hypothesis 1. Further, we found support for Hypothesis 2 as well, professional identity correlated positively with job embeddedness (*r* = 0.194; *p* < 0.01). Unfortunately, we did not find re-entry shock to be significantly related to job embeddedness (*r* = -0.133; *p* > 0.05). Hence, we conducted path analysis between re-entry shock and job embeddedness using professional identity as a mediating variable.

### Examining the Mediation Model

Hypothesis 3 states that professional identity will mediate the relationship between difficulty in re-entry adjustment and job embeddedness. To test for this mediation effect, we followed the approach recommended by Preacher and Hayes (2008). We conducted a mediation analysis using bootstrapping procedures simultaneously as suggested by Preacher and Hayes with the PROCESS macro in SPSS (Hayes, 2013; Preacher and Hayes, 2008).

By adopting the bootstrapping method, we avoid the problem of non-normality in sampling distribution (Preacher and Hayes, 2008). They suggest that a mediation effect can be said to have occurred when the product of the paths between the independent variable and the mediator (path a) and between the mediator and the dependent variable (path b) is statistically significant. Further, the indirect effect of the independent variable should be significant (i.e., zero does not occur between LL and UL) through a bootstrapping test.

The bootstrapping result in **Table 3** shows that the product of path (a) between re-entry shock and professional identity (95% CI = -0.172, -0.038; *p* = 0.002) and path (b) between professional identity and job embeddedness (95% CI = 0.067,0.444; *p* = 0.008), both are statistically significant. The results confirmed the mediation models, suggesting that professional identity mediated the effects of re-entry shock on job embeddedness. Both product of path (a) and path (b) also confirmed Hypothesis 1 and Hypothesis 2 as well as the simple correlation analysis above.

**Table 3:** Coefficients for the mediating effect.

Testing path	Unstandardized Coefficients		*t*	Sig.	Bootstrapping	
	Coefficient	Std. Error			LLCI	ULCI
IV → M (a)	-0.105	0.034	-3.078	0.002	-0.172	-0.038
M → DV (b)	0.256	0.095	2.679	0.008	0.067	0.444
IV → M → DV (c′)	0.016	0.045	0.367	0.714	-0.072	0.104
IV → DV (c)	-0.010	0.044	-0.237	0.813	-0.098	0.077
Indirect effect	-0.027	0.013			-0.055	-0.006

*^a^IV (Re-entry Shock); M (Professional Identity); DV (Job Embeddedness). ^b^All analyses controlled for the length of stay abroad and length of stay back in home country. ^c′^(Direct Effect); ^c^(Total Effect)*.

**FIGURE 1:**
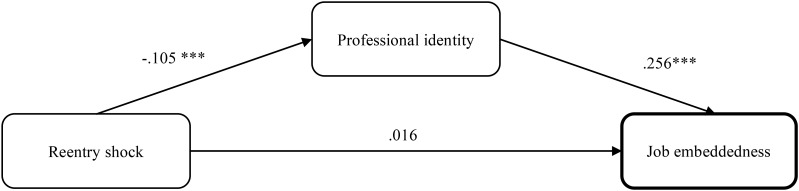
Path diagram for the mediation model.

After controlling for the returnees’ length of stay abroad and length of stay back in the home country, simple mediation models were examined by using PROCESS v3.0 in SPSS, Model 4, and the bootstrapping result indicated that an indirect effect of re-entry shock and job embeddedness via professional identity was significantly negative (Effect = -0.027; *SE* = 0.013; 95% *CI* = -0.055, -0.006). Specifically, there wasn’t a significant total effect (Effect = -0.010; *p* = 0.813) and direct effect (Effect = 0.016; *p* = 0.714) that indicated perfect mediation model. **Figure 1** and **Table 3** present these findings. The negative direction of this relationship suggests that higher level of re-entry shock is associated with lower level of job embeddedness via a sense of professional identity. Thus, Hypothesis 3 is supported by our data. This mediation model also shows that the length of stay abroad (*F* = 1.382; *p* = 0.001) and length of stay back in the home country (*F* = 1.150; *p* = 0.004) are effective as control variables.

## Discussion

The study focusing on demographic characteristics linkage with re-entry adjustment process reveals no different result from that of previous reviews (Szkudlarek, 2010). This present study indicates that the difficulties and distress during re-entry adjustment are greater among Indonesian returnees who have characteristics: female, relatively young age, single status, and in their earlier stay back to Indonesia.

Many studies place the time dimension as a predictor of success or failure of the re-entry adjustment process. Repatriates who spend much of their time abroad tend to be more difficult in the process of readjustment when they return to home country (Black and Gregersen, 1991; Black, 1992; Forster, 1994; Gregersen and Stroh, 1997; Suutari and Välimaa, 2002). Identical with the length of time living abroad, the time elapsed since arrival back to home country is also a predictor of the re-entry adjustment process (Gregersen and Stroh, 1997; Vidal et al., 2007).

An interesting finding within the context of Indonesian returnees is that there is, in inter-correlations between variables tested individually, no correlation between length of stay overseas and difficulty on re-entry adjustment. This finding emphasizes that the high level of difficulty in re-entry adjustment is not caused by what happens when a sojourner is abroad, but more because of the condition after they return to Indonesia. In other words, host country variables do not affect the re-entry adjustment process as also unveiled in previous research by Hammer et al. (1998).

We did not find a previous study that discusses the relationship of demographic characteristics with a sense of professional identity. But in this study, we describe that a strong sense of professional identity was found among returnees who possess characteristics such as elderly in age, high educational level, already married, and longer stay back in the home country after completing study or assignment overseas. Getting older and having responsibilities in the family becomes an indicator of the maturity of persons who are more focused in their field of work and pursue their career. Also, it makes sense if the higher level of education makes them more professional in their profession.

The characteristics of returnees, which are not so different from previous studies, also look at the decision of the returnees to stay in their home country organization. In some reasons, elder employees tend to be more embedded to organization, because it is difficult to find a job after the middle age; and females are less embedded to organization companies, because in general they not career oriented (Peltokorpi, 2013).

We also found that the time dimension also determines the decision to stay in the organization, the longer returnees stay abroad and the longer they have returned to the home country, the higher the decision to be embedded in the recent home country organization. The maturity of returnees and life processes that have been experienced overseas and upon returning to the home country has served as a buffer for commitment to staying in the organization.

The results of this study show that difficulty in adaptation and distress upon coming back to the home country make returnees experience a weak sense of professional identity. The findings confirm previous empirical research that the experience of studying and living abroad contributes to the development of professional identity (Jung et al., 2013; Valk et al., 2013; Jackson, 2015). Experience during living overseas served to broaden cultural, personal, and professional identities, and also the excellent situation for learning and professional development; consequently the identity shifters aspired to new challenges upon re-entry adjustment (Suutari, 2003; Nery-Kjerfve and McLean, 2006; Kohonen, 2008). Kelleher et al. (2016) firmly stated that one of the potential benefits of study abroad is an enhanced professional identity. Furthermore, we opine that professional success is essential to one’s career, but when people fail, they feel their life has no meaning. Distress and negative feeling caused by failure to adapt when one is back in the home country generate a negative interpretation of their own potential possessed and becomes a source of a weakening sense of professional identity.

Furthermore, we analyze the relationship between a sense of professional identity and job embeddedness. As hypothesized, perceived returnees’ professional identity will be associated with job embeddedness, and this was confirmed by the study result. Returnees’ strong sense of professional identity related with high job embeddedness. Indirectly, the findings support previous research that professional identity predicts job-leaving inclinations (Hofman and Kremer, 1985).

The international experience usually accompanying personal changes and organizational and community expectations when a sojourner returns to the home country after living overseas plays a role in their readjustment. When employees go overseas to study or on assignment, they know how valuable they are to the organization, having a bright future in the organization. However, when they are back to their home country, the deal may have changed. Experiences obtained when in overseas bring about changes in returnees’ behavior and mindset related to future career, whether to stay on the job or leave work. In the re-entry issues, if returnees stay back in the home country organization but they un-adjust and un-conform with organizational climate, their performance will decrease and lead to an intention to leave the job (Black et al., 1992; Stroh et al., 1998).

Marsico (2012) states that professional identity is one of the components involved in the process of change in occupational contexts. In line with this, Canrinus et al. (2011) claims that occupational commitment related to the sense of professional identity plays an important role in people’s work environment and daily lives. Professional identity in situations of change, that is, migration from the host country back to the home country, implied an adaptation processes, which most often is stimulated by organizational changes.

Furthermore, we place professional identity as a mediating variable in this study, and the result showed that changes in the sense of professional identity mediated the effect of difficulties in re-entry adjustment on job embeddedness. The flow to make returnees embedded in the organization requires they should be successful on re-entry adjustment process first. Success on re-entry adjustment will increase the sense of professional identity and thus make returnees embedded in their organizations. This result is in line with Marsico’s (2012) statement that professional identity plays a key mediation and modulation role in processes of change in the broader sociocultural context.

The results of this study are identical to the integrative model proposed by Lo Presti and Pluviano (2016), which found human-social capital as an antecedent of job-career orientation with employability as an effective mediator. Lo Presti and Pluviano (2016) place professional development, which in fact is a valuable asset to create a professional identity, as one of the leading components of employability. In future studies, besides professional identity, it is important to pay attention to the role of employability as a mediation variable between re-entry shock and job embeddedness.

The role of the length of stay abroad and length of stay in the home country in this mediation model confirms the results of previous research that have found time dimension to be a predictor of re-entry adjustment process, especially in work-related readjustment models (Black and Gregersen, 1991; Gregersen and Stroh, 1997; Vidal et al., 2007).

## Conclusion

A key finding in this study is that professional identity is an effective mediating variable to bridging the relationship between the phenomenon of post-return conditions to home country with work atmosphere. Professional identity is like a cell membrane that serves as a filter, sensitive, and reactive to the social environment, and also generates adaptation responses in the job or organizational context (Marsico, 2012). We also opine that academic and social activities, when in overseas contributes to the development of returnees’ professional expertise and a better understanding of the organization’s global competitiveness.

Organizations should pay particular attention to their members who have completed studies abroad, one of which can be started by improving the sense of professionalism of the members of the organization. This can help returnees to adapt well after returning from abroad, thus making it less stressful. Attention to this by the organization can automatically make employees feel comfortable, committed to the organization, and finally stay in the organization.

### Study Limitations

Our study’s limitation is found in the sample. This research was exclusively based on a sample of Indonesian returnees from the various professions. Therefore, we recommend conducting this study using a specific profession and similar research in other countries. We also recommend that future research also consider other countries with different levels of economic growth and social life, to increase the chance of generalizing and verifying the results.

The majority of participants in the present study had lived overseas around one year or less (40.4%). This composition may be the reason why in the test of inter-correlations between variables, the length of time living overseas was not related to re-entry shock and to job embeddedness. In future studies, it would be interesting to use a moderated-mediational analysis where the length of stay abroad and length of stay back in home country might moderate the mediational link in the work-related readjustment model, and also control the length of participants’ current job.

### Theoretical Implications

Exiting theories have established the relationships between re-entry adjustment and perceived sense of professional identity, and sense of professional identity and job embeddedness. However, the relationship between returnees’ re-entry adjustment and job embeddedness has remained insignificant. Theoretically, this study has found that a sense of professional identity is the linking variable between re-entry adjustment and job embeddedness; we have found the insignificant relationship between re-entry adjustment and job embeddedness to be explained by the mediating role of sense of professional identity. The results of this study also enrich the reference and empirical evidence of Indonesian returnees’ phenomenon, which has been very limited.

### Practical Implications

As practical implications of the study, results could be useful to plan and design effective post-arrival programs for returnees. At the organizational level, effective post-arrival programs aimed at enhancing professionalism and employability of returnees, which can bring about employee loyalty and embeddedness in the home country organization. Investment in developing professional knowledge and skill for an employee is costly. However, it is essential to do so as it is a crucial focal point for investment in improving organizational performance. From the results of this study, it is again recommended that organizations consider the employee commitment content and a sense of professional identity as one of the content of pre-departure and post-arrival training program.

Further, for the government in home country, the result could be useful to design social policies for returnees, as a national talent, to have moral and social responsibilities to the home country; national social responsibility (NSR). Investment in professional knowledge and skill is also recommended to improve the quality of human resources of a country.

## Author Contributions

SA devised the study and the main conceptual ideas, and is the leading author of this study. MJ supervised the study. All authors listed have made a substantial, direct, and intellectual contribution to the work, and approved it for publication.

## Conflict of Interest Statement

The authors declare that the research was conducted in the absence of any commercial or financial relationships that could be construed as a potential conflict of interest.
